# Circulating metabolites in patients with chronic heart failure are not related to gut leakage or gut dysbiosis

**DOI:** 10.1371/journal.pone.0331692

**Published:** 2025-09-08

**Authors:** Andraž Nendl, Sajan C. Raju, Peder R. Braadland, Anna Nordborg, Vibeke Bratseth, Kaspar Broch, Silje F. Jørgensen, Pål Aukrust, Karsten Kristiansen, Johannes R. Hov, Marius Trøseid, Ayodeji Awoyemi

**Affiliations:** 1 Oslo Center for Clinical Heart Research, Department of Cardiology Ullevaal, Oslo University Hospital, Oslo, Norway; 2 Institute of Clinical Medicine, Faculty of Medicine, University of Oslo, Oslo, Norway; 3 Research Institute of Internal Medicine, Oslo University Hospital Rikshospitalet, Oslo, Norway; 4 Norwegian PSC Research Center, Department of Transplantation Medicine, Oslo University Hospital Rikshospitalet, Oslo, Norway; 5 Department of Biotechnology and Nanomedicine, SINTEF Industry, Trondheim, Norway; 6 Department of Cardiology Rikshospitalet, Oslo University Hospital, Oslo, Norway; 7 Section of Clinical Immunology and Infectious Diseases, Oslo University Hospital Rikshospitalet, Oslo, Norway; 8 Qingdao Key Laboratory of Marine Genomics and Qingdao-Europe Advanced Institute for Life Sciences, BGI Research, Qingdao, China; 9 Laboratory of Genomics and Molecular Biomedicine, Department of Biology, University of Copenhagen, Copenhagen, Denmark; 10 BGI Research, Shenzhen, China; 11 Section of Gastroenterology, Department of Transplantation Medicine, Oslo University Hospital, Oslo, Norway; 12 Department of Cardiology Ullevaal, Oslo University Hospital, Oslo, Norway; South China Agricultural University, CHINA

## Abstract

**Background:**

The gut microbiota produces numerous metabolites that can enter the circulation and exert effects outside the gut. Several studies have reported altered gut microbiota composition and circulating metabolites in patients with chronic heart failure (HF) compared to healthy controls. Limited data is available on the interplay between dysbiotic features of the gut microbiota and altered circulating metabolites in HF patients. We aimed to examine differences in circulating metabolites between people with and without chronic HF, and their association with gut microbiota dysbiosis and cardiac function.

**Methods:**

We collected plasma, serum, and stool samples from 123 adult patients with stable chronic HF and left ventricular ejection fraction (LVEF) ≤40%, and healthy controls (plasma: n = 51, stool samples: n = 69). Metabolomic and lipidomic profiling of plasma was performed using liquid chromatography with tandem mass spectrometry. Principal component analysis was used to explore differences in circulating profiles. Over-representation analysis was performed to identify pathways in which relevant metabolites were involved. Stool samples were sequenced using shotgun metagenomics. We calculated a dysbiosis index based on differential abundances of microbial taxa in patients vs. controls.

**Results:**

After adjusting for age, sex, and sampling location, we identified 67 enriched metabolites and 24 enriched lipids, and 115 depleted metabolites and 6 depleted lipids in HF patients compared to healthy controls. LVEF, N-terminal pro B-type natriuretic peptide, gut leakage markers, dysbiosis index, and fiber intake were not significantly related to any of the differentially abundant metabolites or lipids. Pathways related to energy metabolism differed most between HF patients and controls, however medication adjustment abolished all differences in circulating profiles.

**Conclusions:**

Patients with chronic HF had distinct metabolomic and lipidomic profiles and energy metabolism differed significantly compared to healthy controls before adjusting for medication use. However, the alterations were not related to gut dysbiosis, gut leakage markers, cardiac function, or fiber intake.

## Introduction

While advances in our understanding of the pathophysiology of chronic heart failure (HF) have improved clinical outcomes, it remains a significant burden on healthcare systems worldwide [1]. To better understand the underlying mechanisms of the disease, we need to identify novel biomarkers and therapeutic targets. The gut-heart axis provides a new source of promising candidates [2].

The composition of the gut microbiota differs between patients with HF and healthy individuals [3–5]. The gut microbiota of patients with HF has reduced abundance of important symbiotic bacteria, increased abundance of pathobionts, and has less microbial diversity overall. Together, these features are termed gut dysbiosis. Functionally, the dysbiotic gut has reduced bacterial capacity to produce short-chain fatty acids (SCFAs), which are fermentation products of dietary fiber, associated with improved host health [3,4].

Several bacterially produced metabolites beyond SCFA have received attention as potential pathogenic factors and disease modifiers in HF [2]. For instance, the diet-dependent metabolite trimethylamine-N-oxide (TMAO), secondary bile acids, and amino acid derivatives such as imidazole propionate, indoxyl sulfate, and phenylacetylglutamine have been implicated in the pathogenesis of HF [6–9]. In HF, intestinal hypoperfusion and congestion results in gut barrier dysfunction [10]. This leads to leakage of bacteria or bacterial products such as lipopolysaccharide (LPS) into the bloodstream with a subsequent innate immune response through interaction with LPS-binding protein (LBP), cluster of differentiation 14 (CD14), and Toll-like receptor 4 [11].

Energy utilization is disrupted in the failing heart, which is reflected in altered profiles of circulating metabolites [12,13]. The main driving force behind variation in circulating metabolites in healthy people is, apart from diet, the gut microbiota [14]. Although some research has been performed on the connection between circulating metabolites/lipids and the gut microbiota in chronic HF [15], exact mechanisms and therapeutic targets remain elusive. So far, one large study has studied this relationship in cardiometabolic diseases and ischemic heart disease, including HF [16].

In this cross-sectional, exploratory study, we aimed to 1) examine differences in circulating metabolites between patients with chronic HF and healthy controls, 2) examine associations between circulating metabolites and gut microbiota alterations including markers of gut leakage, and 3) investigate associations between circulating metabolites and cardiac function.

## Materials and methods

### Study participants/cohorts

This study uses the baseline data for the Norwegian participants of the randomized GutHeart trial (n = 123). Participants were recruited between 11 March 2016 and 20 June 2019 from three sites: Oslo University Hospital Ullevål, Oslo University Hospital Rikshospitalet, and Nordland Hospital Bodø. A comprehensive list of inclusion and exclusion criteria has been published [17]. Briefly, inclusion criteria for subjects with HF required symptomatic HF with a left ventricular ejection fraction (LVEF) of ≤40%, New York Heart Association (NYHA) functional class II–III, and a history of optimal medical treatment within the three months preceding study inclusion. Two participants were excluded for missing either metabolomic or lipidomic data.

We included plasma samples from 51 healthy controls and stool samples from a separate cohort of 69 healthy controls. The control subjects in both cohorts were in good health and did not use medications on a regular basis. None of the study subjects had used antibiotics or probiotics in the three months leading up to sampling. Their data were accessed on 15 March 2024. Authors did not have access to information that could identify individual control subjects during or after data collection. The research adhered to the principles outlined in the Declaration of Helsinki. The Norwegian Medicines Agency and the Regional Ethics Committees of South-East Norway (reference numbers 2015/120 and 2019/292, REK sør-øst) approved the study. The GutHeart trial was registered at ClinicalTrials.gov (NCT02637167). Written informed consent was obtained from all participants prior to their involvement in the study.

### Stool samples for metagenomic analyses

Stool samples from the HF cohort and healthy controls were collected in tubes with a DNA-stabilizing solution (Stratec Molecular GMBH, Berlin, Germany). Fecal DNA was extracted using the PSP Spin Stool DNA kit (Stratec Molecular GMBH). The samples were analyzed by shotgun sequencing on the MGISEQ-T7 platform. The generated reads were then processed as previously published [18].

### Blood sampling protocol

Before blood sampling, all participants had fasted overnight. Serum and EDTA-plasma were separated by centrifugation at room temperature and at 4°C, respectively, within one hour of collection. The collected sample materials were stored at −80°C. The same blood collection protocol was used for subjects with HF and healthy controls.

### Analysis of cardiac function and gut leakage markers

Transthoracic echocardiography was performed and LVEF determined using the modified Simpson’s rule, as detailed in the main study [17].

Levels of N-terminal pro B-type natriuretic peptide (NT-proBNP) were determined in serum using an electrochemiluminescence immunoassay (ECLIA) as a component of routine clinical care, utilizing reagents from Roche Diagnostics, Mannheim, Germany.

Markers of gut leakage LPS, LBP, soluble CD14 (sCD14), and intestinal fatty acid binding protein (I-FABP) were analyzed in serum as previously described [19].

### Untargeted metabolomics and lipidomics analysis

The untargeted metabolomics and lipidomics analyses were performed by high performance liquid chromatography (Agilent 1290, Agilent, Santa Clara, CA, USA) coupled to high resolution Bruker Impact II quadrupole time-of-flight (Q-TOF) mass spectrometer (Bruker Daltonics, Bremen, Germany). Preparation of plasma samples was performed following two different procedures yielding three different sample extracts for each patient sample: i) protein precipitation and extraction by addition of four volumes of cold methanol, followed by centrifugation at 14.000 G and collection of the resulting supernatant for analysis. ii) an in-house modified SIMPLEX (Simultaneous Metabolite, Protein, Lipid Extraction) extraction resulting in a hydrophilic sample for metabolomics analysis and a lipophilic sample for lipidomics analysis [20]. To each 100 µl of plasma, 225 µl of ice-cold methanol were added, followed by vortex mixing for 10 sec and addition of 750 µL of ice-cold methyl tert-butyl ether followed by another vortex for 10 seconds. Samples were shaken for 5 minutes prior to addition of 400 µl water and another vortex for 20 seconds. After centrifugation at 14.000 G for 2 minutes, 400 µl of the upper lipophilic phase was transferred to 300 µl micro inserts and evaporated to dryness. Remaining lipophilic phase was removed prior to addition of 100 µl chloroform and a 10 second vortex. A 250 µl portion of the upper hydrophilic phase was collected and evaporated to dryness. The lipophilic phase was re-solubilized in methanol:toluene 9:1 and the hydrophilic phase was solubilized in methanol. The lipophilic phase was subjected to lipidomics analysis in positive ionization mode utilizing a Waters BEH C18 (2.1 x 50 mm, 2.7um, Waters, Milford, MA, USA) column using gradient elution with 10 mM ammonium formate with 0.01% formic acid and 30–100 per cent acetonitrile:isopropanol 1:1 as organic modifier. The hydrophilic phase sample was analyzed using two different metabolomics methods, a HILIC (hydrophilic interaction liquid chromatography) method employing a SeQuant ZIC-HILIC (2.1x150 mm, 3.5, 100Å, Merck Millipore, Darmstadt, Germany) column for analysis of highly hydrophilic metabolites and a reversed-phase mode analysis (RP) on a Waters Aquity HSS T3 (2.1 x 100 mm, 1.8 µm, Waters, Milford, MA, USA) column. The sample subjected to protein precipitation by methanol addition was analyzed using a slight modification of the lipidomics method. HILIC analysis utilized gradient elution with 10 mM ammonium acetate in 90–10% acetonitrile while RP analysis was performed employing 5 mM ammonium formate and 0.1% formic acid with 5–95% methanol for gradient elution. Analysis was performed using electrospray ionization, in negative ionization mode for the HILIC analysis and positive ionization mode for the RP metabolomics and the lipidomics analysis. Mass spectrometric data was acquired utilizing a data dependent acquisition method with cycle time of 0.5 seconds.

Feature extraction was conducted utilizing Bruker Metaboscape software (Bruker Daltonics, Bremen, Germany).

### Liquid chromatography with tandem mass spectrometry spectrum processing and metabolite annotation

We used the MetaboAnalyst 4.0 (available online and in R) pipeline to preprocess and filter the raw spectra [21]. Interquartile range (IQR) filtering was used to remove variables (metabolites) with low variability, which are often non-informative. The IQR measures the spread of the middle 50% of data for each variable. Metabolites with an IQR below a set threshold are filtered out, leaving only those with higher variability for further analysis. This helps focus on biologically relevant patterns and reduces noise. Sample normalization by median was applied to adjust for systematic differences among samples. Data underwent log transformation, and auto-scaling was used to adjust each variable by a scaling factor based on its dispersion.

Metabolites were putatively identified using the R package metID. This package matches individual peak mass to charge ratios and tandem mass spectrometry spectra with public metabolomics databases [22].

### Food frequency questionnaires

Patients completed a validated Norwegian food frequency questionnaire, that aims to assess dietary habits over the past year. Fiber intake, expressed in grams, was calculated from the questionnaire responses using software developed at the Institute for Nutrition Research, University of Oslo.

### Statistical analyses

Principal component analysis (PCA) was performed using MetaboAnalyst 4.0 on quality control-filtered data. This allowed for an examination of groupings, trends (both similarities and differences within and between sample groups), and identification of outliers within the dataset based on the observed variables.

We used the microbial dysbiosis index (adjusted for age, sex and body mass index), calculated for each sample as log_10_ (sum of the abundances of the bacterial species increased in HF/sum of the abundances of species decreased in HF), as described in [23].

We used multivariable logistic regression to estimate each metabolite’s age- and sex-adjusted association with HF status (healthy controls as reference). To explore whether specific metabolic pathways differed significantly in HF, we used MetaboAnalyst’s over-representation analysis (ORA). We considered three separate inputs to the ORA, namely using (1) all differently abundant metabolites, (2) exclusively more abundant metabolites, and (3) exclusively less abundant metabolites.

We performed a correlative analysis between each annotated, enriched or depleted metabolite and the dysbiosis index, markers of gut leakage (LPS, LBP, sCD14, and I-FABP), and cardiac function (LVEF and NT-proBNP). The strength of bivariate associations was tested using Spearman’s correlation analyses. We adjusted for multiple testing using the Bonferroni correction, with an adjusted alpha of 2·10^-5^ for 2134 correlation analyses.

For sensitivity analyses, we performed Spearman’s correlation analyses between differentially abundant metabolites and fiber intake, using false discovery rate correction for multiple testing. Additionally, we included medication as covariates in the abovementioned multivariable logistic regression models to examine their effect on circulating metabolomic and lipidomic profiles. Statistical analyses were performed in R v. 4.3.2 and Python 3.8.5.

## Results

Clinical characteristics for the patients with HF and the healthy controls are given in Table 1. Of note, the HF population had more men and was younger than the healthy controls and this has been adjusted for in later analyses. Within the HF cohort, the majority had HF of ischemic etiology, NT-proBNP was modestly elevated, and patients were optimally treated with high adherence to contemporary guideline-recommended medications.

**Table 1:** Demographic and clinical characteristics of study participants.

Characteristics	Heart failure	Healthy controls	
		Plasma samples	Stool samples
	n = 123	n = 51	n = 69
Age, years	61.6 ± 8.7	67.8 ± 6.9^*^	51.7 ± 3.7^*^
Female sex	24 (20%)	22 (43%)^*^	35 (51%)^*^
BMI, kg/m^2^	28.7 ± 4.9	24.9 ± 3.6^	25.6 ± 3.3
NYHA class			
Class II	94 (76%)		
Class III	29 (24%)		
**Medical history**			
Hypertension	41 (36%)		
Diabetes mellitus	30 (26%)		
Current smokers	49 (40%)		
Heart failure etiology			
Ischemic heart failure	72 (59%)		
Non-ischemic	51 (41%)		
History of PCI and/or CABG	62 (50%)		
**Medication use**			
ACEi or ARB	120 (98%)		
Beta-blocker	121 (98%)		
Mineralocorticoid receptor antagonist	72 (59%)		
Neprilysin inhibitor	18 (15%)		
Loop diuretics	76 (62%)		
**Markers of cardiac function**			
NT-proBNP, pg/mL	1150 [461, 1720]		
LVEF, %	31 [24, 35]		
**Gut leakage markers**			
LPS, pg/mL	30.8 [24.7, 36.5]		
LBP, ng/mL	19645 [16133, 22818]		
I-FABP, pg/mL	1432 [800, 2233]		
sCD14, ng/mL	1366 [1190, 1588]		
**Other relevant measurements**			
CRP, mg/L	1.5 [0.7, 3.2]		
Creatinine, µmol/L	95 [81, 117]		
Microbial dysbiosis index	−3.9 [−5.1, −2.6]		

Continuous variables are given as mean ± standard deviation or median quartile 1, quartile 3. Proportions are given as n (%),

* p < 0.001 using Wilcoxon rank-sum test (age and BMI) and Fisher’s exact test (sex) for differences between each healthy cohort and heart failure cohort,

^n = 13 for BMI in healthy controls for plasma samples. BMI – body mass index; NYHA – New York Heart Association; PCI – percutaneous coronary intervention; CABG – coronary artery bypass graft; NT-proBNP – N-terminal pro-B-type natriuretic peptide; LVEF – left ventricular ejection fraction; LPS – lipopolysaccharide; LBP – LPS-binding protein; I-FABP – intestinal fatty acid binding protein; sCD14 – soluble cluster of differentiation 14; CRP – C-reactive protein.

### Metabolomic and lipidomic profiling of heart failure patients and healthy controls

**Fig 1:**
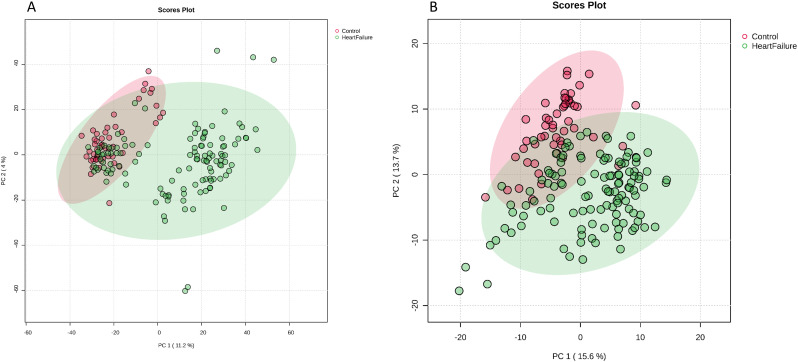
Principal component analysis plots of heart failure patients and healthy controls. **Plots in A) metabolomes and in B) lipidomes.** One outlier excluded for metabolomes. Figure with outlier included available as S1 Fig.

To investigate the metabolic differences between the HF cohort and the healthy controls, we used LC-MS/MS for metabolite and lipid profiling. We identified 16,697 distinct metabolites and 366 lipids. One control sample was excluded due to their classification as an outlier following a multivariable PCA (Fig 1A and 1B).

#### Metabolomic profiling.

**Fig 2:**
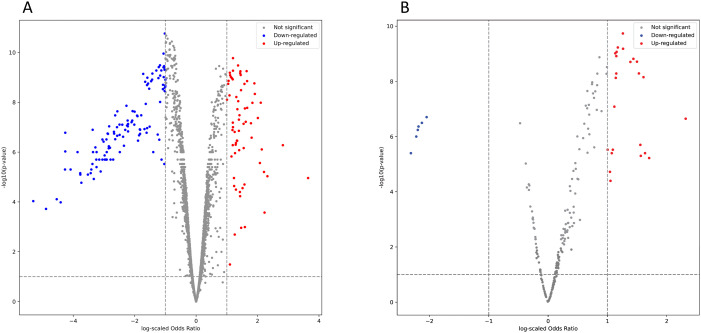
Volcano plots of A) metabolites and B) lipids. Enriched metabolites/lipids in red and depleted in blue.

Initial quality control procedures yielded a dataset comprising of 5000 metabolites. As age and sex differed between the patients with HF and healthy controls, and both variables are known to markedly confound metabolomic profiles, we adjusted for age and sex in a logistic regression model [24]. Additionally, we observed a notable clustering of patients included at one center (Bodø) with the healthy controls. We therefore added sampling location to the model and reanalyzed the data. A total of 1084 metabolites were differentially abundant in the HF cohort versus the healthy controls. At an alpha threshold of 0.05, 67 metabolites were more abundant in the HF cohort than in the healthy controls with a log odds ratio ≥1. On the other hand, 115 metabolites were less abundant in patients with HF than in the healthy control subjects (log odds ratio ≤ −1; Fig 2A). We successfully annotated 54 enriched metabolites (S1 Table) and 111 depleted metabolites (S2 Table). The annotated metabolites did not include the aforementioned TMAO, imidazole propionate, indoxyl sulfate, phenylacetylglutamine, or secondary bile acids.

**Fig 3:**
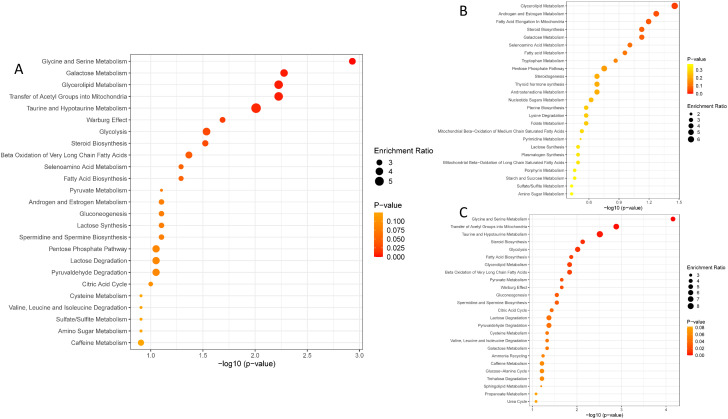
Summary of over-representation pathway analyses. Based on A) all differentially abundant metabolites, B) enriched metabolites and C) depleted metabolites.

The metabolites that differed in abundance between patients with HF and controls, were involved in pathways such as glycine and serine metabolism, galactose metabolism, glycerolipid metabolism, taurine and hypotaurine metabolism, pathways involved in the Warburg effect, and glycolysis. Based on only enriched metabolites, the most significant pathways were glycerolipid metabolism, androgen and estrogen metabolism, and fatty acid elongation in mitochondria. Conversely, pathways such as glycine and serine metabolism, transfer of acetyl groups into mitochondria, and taurine and hypotaurine metabolism, were the most significant based on only depleted metabolites in the HF cohort (Fig 3).

#### Lipidomic profiling.

After initial quality control procedures, we compiled a dataset of 219 lipids. After adjustment for age, sex, and sampling location, we identified 133 differentially abundant lipids in the HF cohort versus healthy controls. With the threshold set at log odds ratio ≥1 and ≤−1, we found 24 more abundant lipids, and six less abundant lipids in the HF cohort than in the healthy controls, respectively (Fig 2B). We successfully annotated 23 enriched lipids (S3 Table), and all depleted lipids (S4 Table).

**Fig 4:**
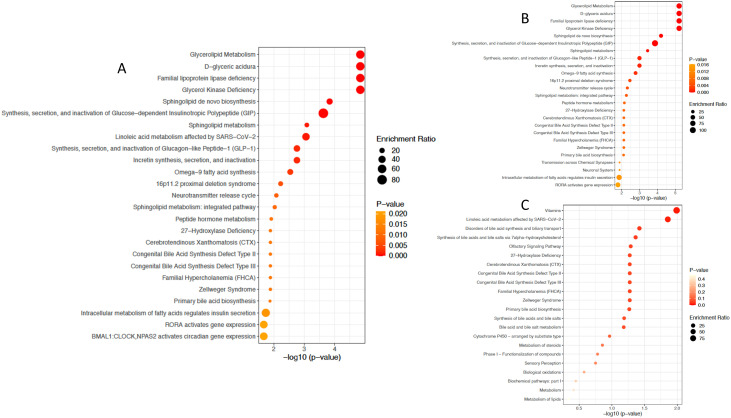
Summary of over-representation pathway analyses. Based on A) all differentially abundant lipids, B) enriched lipids and C) depleted lipids.

The lipids that differed in abundance in patients with HF compared with healthy controls were involved in glycerolipid metabolism, sphingolipid de novo synthesis and metabolism, synthesis, secretion, and inactivation of glucose-dependent insulinotropic peptide, glucagon-like peptide 1, and incretin. Based on only enriched lipids, the most significant pathways were essentially the same as the ones based on all differentially abundant lipids. Conversely, the lipids that were the most depleted in patients with HF versus healthy controls come from pathways involving vitamins, linoleic acid metabolism, and bile acid synthesis and biliary transport (Fig 4).

### Metabolomics/lipidomics and gut microbiota-related parameters

**Fig 5:**
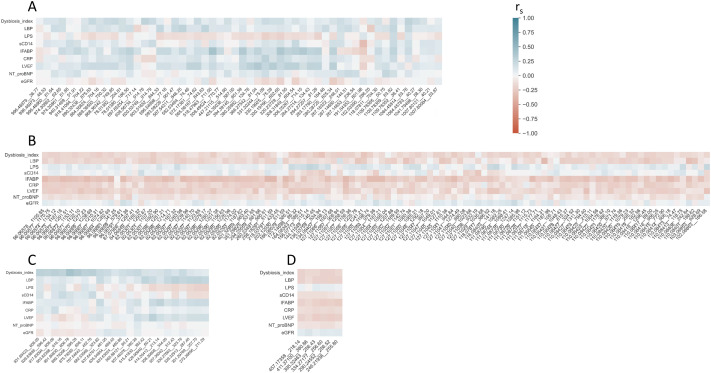
Heat maps of correlation analyses. **Correlations between A) enriched metabolites, B) depleted metabolites, C) enriched lipids, and D) depleted lipids, and the dysbiosis index, gut leakage markers and cardiac function.** The numbers on the x-axes show the mass and charge of individual metabolite/lipid in the format of “mass__charge”. The annotation for metabolites and lipids listed on the x-axes can be found in S1–S4 Tables. The p-values and values of Spearman’s correlation coefficient are given in S1 File. CRP – C-reactive protein; eGFR – estimated glomerular filtration rate; I-FABP – intestinal fatty acid binding protein; LBP – lipopolysaccharide binding protein; LPS – lipopolysaccharide; LVEF – left ventricular ejection fraction; NT-proBNP – N-terminal pro-B-type natriuretic peptide; sCD14 – soluble cluster of differentiation 14; r_s_ – Spearman’s correlation coefficient.

We found weak negative correlations between the dysbiosis index and 11 depleted metabolites. These associations disappeared after correction for multiple testing (Fig 5). Furthermore, of the gut leakage markers, LPS was weakly positively correlated to four depleted metabolites, LBP weakly negatively to 16 depleted metabolites, sCD14 weakly negatively to three depleted metabolites, and I-FABP weakly negatively to 80 depleted metabolites. The correlations did not withstand correcting for multiple testing (S1 File). None of the differentially abundant lipids were significantly correlated with the dysbiosis index or gut leakage markers.

### Metabolomics/lipidomics and cardiac function

We found weak negative correlations between LVEF and 35 depleted metabolites, and NT-proBNP and one depleted metabolite. Statistical significance was lost after correcting for multiple testing, suggesting that the traditional markers of cardiac function may not directly align with metabolic alterations observed in HF (Fig 5, S1 File). None of the differentially abundant lipids were significantly correlated with LVEF or NT-proBNP.

### Metabolomics/lipidomics and fiber intake

We performed correlation analyses between the differentially abundant metabolites or lipids and fiber intake and found no statistically significant associations (data not shown).

### Metabolomics/lipidomics and medication

To assess the impact of medication on circulating metabolites and lipids, we included renin-angiotensin system (RAS) inhibitors, angiotensin receptor/neprilysin inhibitors (ARNIs), mineralocorticoid receptor antagonists (MRAs), loop diuretics, and beta blockers as covariates in logistic regression models in addition to the aforementioned age, sex, and sampling location. We found that after inclusion of RAS inhibitors, ARNIs, MRAs, and loop diuretics, 10 enriched metabolites remained statistically significant (S2 File). After including also beta blockers in the analysis, none of the metabolites or lipids remained significant.

## Discussion

In this study of untargeted metabolomics and lipidomics in HF, we aimed to explore differences in circulating metabolites between patients with chronic HF and healthy controls, and to examine whether the differentially abundant metabolites relate to gut dysbiosis and gut barrier dysfunction. Previous studies have identified extensive differences in the circulating metabolomic and lipidomic profiles between subjects with and without HF [13,25]. Additionally, subjects with HF have demonstrated gut microbiota dysbiosis that increases with disease severity. However, it is unknown whether this gut dysbiosis contributes to the circulating metabolomic profiles.

Unadjusted for medication use, we found that glycine and serine metabolism, galactose metabolism, glycerolipid metabolism, taurine and hypotaurine metabolism, pathways involved in the Warburg effect, and glycolysis, differed the most between HF patients and healthy controls (Fig 3). These results suggest a change in energy substrate preference in HF which is in line with previous reports [12,13]. The most differentially abundant lipids were involved in glycerolipid metabolism, sphingolipid de novo synthesis and metabolism and notably, synthesis, secretion, and inactivation of glucose-dependent insulinotropic peptide, glucagon-like peptide 1 (GLP-1), and incretin.

We used a dysbiosis index to investigate potential associations between the extent of gut dysbiosis and the observed metabolic differences. However, after adjustment for multiple testing, our analysis revealed no significant correlations between the dysbiosis index and the differentially abundant metabolites or lipids. Our findings suggest that the substantial metabolic perturbations in HF patients are largely independent of the degree of gut dysbiosis measured by the dysbiosis index.

Given the lack of a universal definition for dysbiosis, comparing its severity across different studies remains a challenge [26]. Nonetheless, previous research has identified dysbiosis in the context of chronic HF. The extent of dysbiosis appears to be related to the degree of congestion and the symptomatic burden of HF [27]. Our HF cohort consisted of well-treated, stable chronic HF patients. Most were in NYHA class II and had modestly elevated NT-proBNP. More pronounced associations might be observed in a more symptomatic cohort. Furthermore, changes in specific microbial taxa and their specific function might play a more crucial role in the disease process than changes in the overall microbiota composition indicated by the dysbiosis index. As an example, the microbial metabolite TMAO has been identified as both a biomarker and a possible contributor to the pathogenesis of HF [7,28]. Trimethylamine, the precursor to TMAO, can be produced by several bacterial taxa, primarily within the phylum Firmicutes [29]. However, TMAO has not been associated with HF-related dysbiosis in previous studies, in line with the lack of correlation between circulating metabolites and HF-related dysbiosis in this study [30].

The differentially abundant metabolites were not associated with markers of gut leakage. This suggests that the observed metabolic differences between patients with HF and healthy controls are not related to intestinal mucosal damage, leakage of bacterial products into the circulation, or the subsequent immune response [31,32]. LPS, LBP and sCD14 have been linked to the pathogenesis of coronary artery disease and HF [33]. However, our previous findings indicated no association between these markers and cardiac function in the GutHeart population [19]. The current lack of association with metabolic alterations further suggests minimal impact of gut leakage in this population. Although I-FABP was associated with cardiac function in the aforementioned study, the absence of a relationship between I-FABP and metabolic alterations could indicate that I-FABP merely reflects the extent of intestinal mucosal damage without affecting metabolism directly.

The differences in metabolic pathways appear to be unrelated to cardiac function. This is somewhat surprising, as it is thought that differences in HF severity may lead to, e.g., different patterns of fatty acid utilization [34]. A possible explanation could be the clinical stability of our patients, as previously highlighted [35]. However, our study participants are similar to participants in other major HF trials in NYHA class, NT-proBNP levels and comorbidities, and thus typical of contemporary HF patients [36,37]. This study only examined people with HF with reduced ejection fraction, and it has been suggested that inflammation and cardiometabolic disturbances play a greater role in HF with preserved ejection fraction, a disease that is also associated with dysbiosis [38,39].

The majority of differentially abundant metabolites in our study are related to energy utilization. The failing heart is generally understood to reduce its combustion of fatty acids, with a compensatory increase in glycolysis. However, our results suggest increased fatty acid utilization. A similar metabolic shift has been observed in metabolic syndrome, obesity, and type 2 diabetes mellitus. These conditions are thought to lead to either no change or an increase in fatty acid utilization [34]. Our population was on average overweight, and a quarter had diabetes, which may have affected our results. Some intermediates in energy metabolism are also thought to have roles beyond providing fuel for the heart, acting as auto-, para- or endocrine signaling molecules and cofactors in regulating gene expression [40]. Thus, the observed metabolic alterations may have more far-reaching effects than just changed fuel utilization in the heart. Interestingly, the GLP-1 receptor agonist semaglutide has been shown to reduce symptoms and improve exercise capacity in obese patients with HF with preserved ejection fraction, possibly beyond its effects on weight reduction [41]. Taken together with our finding of altered GLP-1 and incretin metabolism in our HF cohort, this suggests a role for incretins in pathogenesis of HF.

Several amino acid-related pathways were significantly different between HF patients and healthy controls. Serine and glycine metabolism has been extensively studied in cancer, where malignant cells increase their biosynthesis to aid unrestricted proliferation [42]. Their metabolism was significantly downregulated in HF patients, potentially suggesting a general state of catabolism. An interesting finding is the depletion of hypotaurine and taurine metabolism in HF patients. Taurine is a major free intracellular amino acid in the heart, where it is present at approximately 100 times higher concentrations than in plasma [43]. It has a plethora of reported beneficial effects, including antioxidant activity, modulation of energy metabolism, gene expression, endoplasmic reticular stress and Ca^2+^ homeostasis [44].

Low fiber intake has previously been associated with gut microbiota alterations in chronic HF, including reduced bacterial diversity and a lower Firmicutes/Bacteroidetes ratio, which in turn correlated with adverse clinical outcomes [45]. In our study, fiber intake was not associated with any of the differentially abundant metabolites or lipids, despite the previous report of a negative correlation between fiber intake and imidazole propionate in the GutHeart population [18].

Commonly prescribed HF medications emerged as the dominant driver for the observed metabolic differences. Sequential adjustment for RAS inhibitors, ARNIs, MRAs, and loop diuretics reduced the enriched metabolites to 10, and the addition of beta blockers eliminated all significant differences between patients and controls. Our results thus suggest that the observed differences in circulating profiles may mainly be due to medication use.

Our study has some limitations. We have limited clinical data and laboratory analyses available in the healthy control subjects. Dysbiosis index calculation was based on stool samples from a cohort of healthy control subjects different from the healthy controls that donated plasma samples for metabolomics and lipidomics analyses. As this was an untargeted metabolomics study without internal standards, the results are subject to several pitfalls in the analysis process [46]. There may be uncertainties related to the exact molecular structure of the metabolites annotated based on retention time and molecular weight. This has been taken into account by focusing on overall assessment of pathways and not highlighting individual metabolites as biomarkers, which should preferably be validated with targeted methods. Storage of plasma samples at −80°C for several years prior to analysis, such as in our case, can alter concentrations of certain metabolites and lipids [47]. All analyses were performed on peripheral blood samples, not from local vessels of the heart or the myocardium itself. Therefore, the findings reflect processes in the entire body, not specifically in the heart.

## Conclusions

In patients with stable chronic HF with reduced LVEF, we found distinct metabolomic and lipidomic profiles compared to healthy controls. Energy metabolism differed significantly between patients with HF and healthy controls. The differences appeared to be largely related to medication use. However, the alterations in circulating metabolites and lipids were not related to gut dysbiosis, gut leakage markers, markers of cardiac function, or fiber intake in our HF cohort.

## Supporting information

S1 Fig Principal component analysis plot of heart failure patients and healthy controls in metabolomes with outlier included. (TIFF)

S1 Table Enriched metabolites annotation. (DOCX)

S2 Table Depleted metabolites annotation. (DOCX)

S3 Table Enriched lipids annotation. (DOCX)

S4 Table Depleted lipids annotation. (DOCX)

S1 File Correlations between differentially abundant metabolites/lipids and gut-related parameters or cardiac function. (XLSX)

S2 File Differentially abundant metabolites/lipids after adjusting for medication. (XLSX)
