# Supplementary figures and images for: Circulating metabolites in patients with chronic heart failure are not related to gut leakage or gut dysbiosis
Source: PLoS One. 2025 Sep 8;20(9):e0331692. doi: 10.1371/journal.pone.0331692 (PMC12416712; doi:10.1371/journal.pone.0331692)

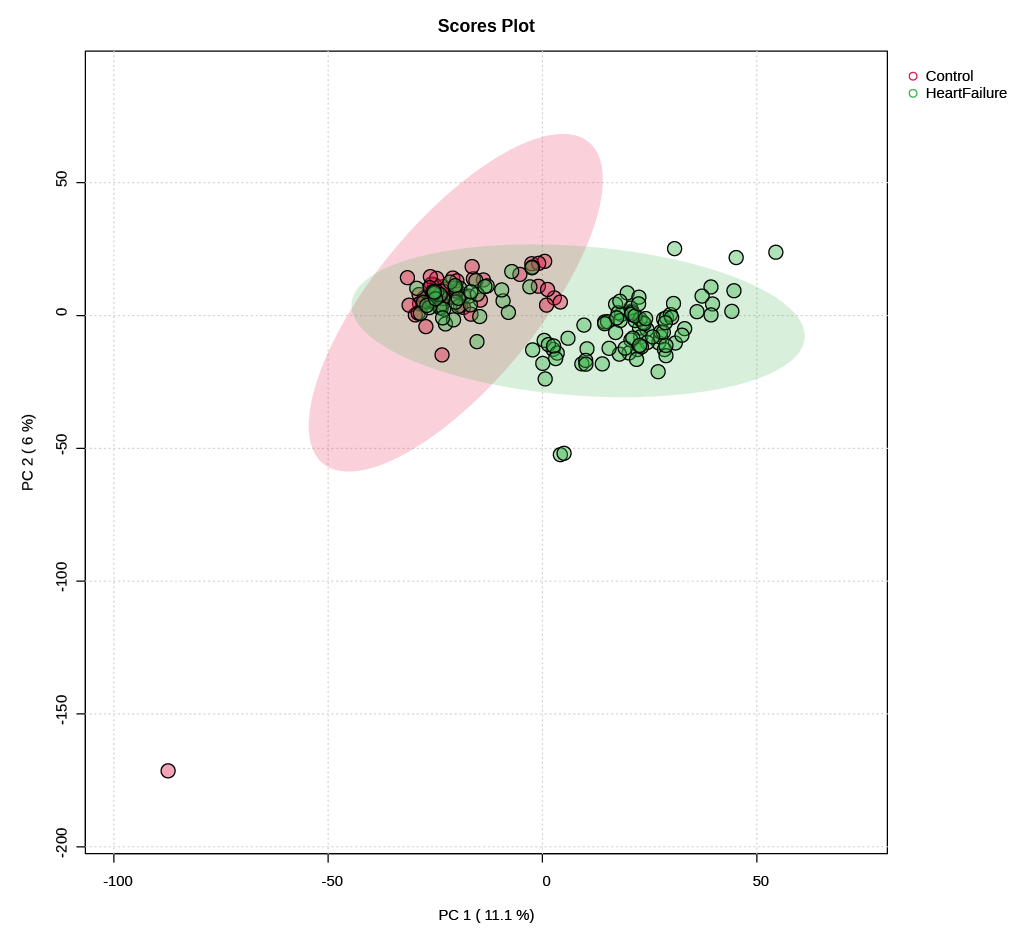

Supplement: S1 Fig — (TIFF) [file pone.0331692.s001.tiff]
