# Supplementary material for: Circulating metabolites in patients with chronic heart failure are not related to gut leakage or gut dysbiosis
Source: PLoS One. 2025 Sep 8;20(9):e0331692. doi: 10.1371/journal.pone.0331692 (PMC12416712; doi:10.1371/journal.pone.0331692)
Supplement: S1 Table — (DOCX) [file pone.0331692.s002.docx]

**S1 Table**. Enriched metabolites annotation.

| Metabolites | Compound name | p-value | Odds ratio | Log(odds ratio) |
| --- | --- | --- | --- | --- |
| 996.46979__36.77 | Congmuyenoside A | 4.28E-08 | 102.45548 | 2.01053519 |
| 996.46973__48.53 | Congmuyenoside A | 5.68E-10 | 27.676156 | 1.44210577 |
| 996.46960__61.64 | Congmuyenoside A | 1.05E-08 | 68.156015 | 1.83350419 |
| 974.30862__62.65 | Dasatinib | 5.28E-09 | 12.210072 | 1.08671822 |
| 974.30861__51.85 | Dasatinib | 1.38E-09 | 11.406126 | 1.05713816 |
| 940.31469__51.91 | 3,4,5-trihydroxy-6-{3-hydroxy-5-[(E)-2-(4-hydroxyphenyl)ethenyl]-2-[(1E)-3-methylbuta-1,3-dien-1-yl]phenoxy}oxane-2-carboxylic acid | 1.81E-09 | 11.093524 | 1.04506953 |
| 918.41064__704.22 | Pregnenolone sulfate | 3.50E-07 | 21.70394 | 1.33653858 |
| 890.32438__875.83 | Cycloartocarpin | 8.38E-07 | 25.20764 | 1.40153219 |
| 884.35824__704.16 | Heme O | 3.45E-07 | 21.316563 | 1.32871718 |
| 869.36593__750.32 | Mangostanol | 2.30E-05 | 17.298037 | 1.23799682 |
| 868.36342__749.85 | beta-D-Xylopyranosyl-(1->4)-alpha-L-rhamnopyranosyl-(1->2)-D-fucose | 1.55E-07 | 17.996927 | 1.25519836 |
| 78.01382__204.81 | Dimethyl sulfoxide | 4.98E-08 | 24.745395 | 1.39349439 |
| 78.01380__186.50 | Dimethyl sulfoxide | 1.10E-05 | 4423.06749 | 3.64572357 |
| 697.65254__717.14 | Cer(d18:0/23:0) | 0.032509 | 12.619108 | 1.10102866 |
| 638.56734__914.76 | DG(20:0/0:0/20:3n9) | 1.67E-08 | 45.999929 | 1.66275716 |
| 620.53749__914.79 | DG(14:0/20:0/0:0) | 9.04E-09 | 23.506103 | 1.37118063 |
| 603.51652__844.33 | 1-[1,4-Dihydro-4-nonyl-5-(1-oxodecyl)-3-pyridinyl]-1-dodecanone | 2.00E-05 | 37.473439 | 1.57372355 |
| 596.54998__77.18 | CE(14:0) | 3.31E-08 | 25.803854 | 1.41168458 |
| 593.59226__901.47 | 5b-Pregnanediol | 0.002046 | 18.092323 | 1.25749433 |
| 587.54311__846.25 | Tridodecylamine | 2.74E-06 | 119.297426 | 2.07663107 |
| 582.53464__74.46 | N-(2R-Hydroxyhexadecanoyl)-2S-amino-9-methyl-4E,8E-octadecadiene-1,3R-diol | 6.90E-07 | 35.387764 | 1.54885312 |
| 572.17381__74.62 | 3-(4-Hydroxy-3-methoxyphenyl)-1,2-propanediol 2-O-(galloyl-glucoside) | 1.00E-07 | 15.847185 | 1.19995213 |
| 565.56167__843.63 | Digeranyl | 6.28E-06 | 162.052983 | 2.20965703 |
| 518.47963__711.00 | 1-Tritriacontanol | 4.30E-07 | 19.80193 | 1.29670752 |
| 508.49524__770.77 | 2,3,4-Trimethyltriacontane | 6.95E-08 | 60.958075 | 1.78503124 |
| 447.21115__514.90 | Terazosin | 2.80E-05 | 32.499436 | 1.51187582 |
| 425.35038__587.00 | MG(0:0/20:1(11Z)/0:0) | 7.85E-09 | 10.122345 | 1.00528114 |
| 394.39145__651.39 | 24-Propylcholestan-3-ol | 1.10E-06 | 18.472125 | 1.26651686 |
| 380.25982__134.76 | Bisnorcholic acid | 5.39E-10 | 15.880641 | 1.20086803 |
| 366.27542__81.04 | 10,20-Dihydroxyeicosanoic acid | 1.77E-08 | 38.65574 | 1.58721399 |
| 331.20244__75.09 | Lotusine | 6.58E-08 | 15.667461 | 1.19499862 |
| 330.19925__75.28 | Bexarotene | 1.30E-07 | 15.258115 | 1.18350088 |
| 330.19766__652.65 | Lysyl-Arginine | 5.06E-07 | 19.417331 | 1.28818953 |
| 320.23729__81.55 | Bisnorcholic acid | 1.90E-08 | 22.046758 | 1.34334474 |
| 308.31849__654.54 | Tripropylamine | 1.50E-06 | 14.168289 | 1.15131741 |
| 306.25267__714.15 | Stearic acid | 1.20E-09 | 15.580579 | 1.19258359 |
| 284.27214__134.43 | (E)-3-Nonen-1-ol | 6.51E-10 | 12.536869 | 1.09818909 |
| 284.27207__81.29 | (E)-3-Nonen-1-ol | 6.08E-08 | 41.340413 | 1.61637481 |
| 283.17760__184.48 | 2-Carboxy-4-dodecanolide | 3.20E-05 | 19.815101 | 1.29699629 |
| 280.28725__625.34 | Decamethonium | 5.26E-07 | 15.596269 | 1.19302072 |
| 267.18322__246.01 | 3-hydroxyoctanoyl carnitine | 0.000268 | 168.983847 | 2.22784519 |
| 267.14680__144.51 | Pimelylcarnitine | 4.00E-05 | 26.654397 | 1.42576886 |
| 267.14653__236.54 | Pimelylcarnitine | 5.90E-05 | 27.095241 | 1.43289302 |
| 191.04353__601.98 | 2,5-Dimethyl-3-(methyldithio)furan | 5.28E-07 | 667.098375 | 2.82418988 |
| 152.03390__286.20 | Xanthine | 0.001097 | 29.265078 | 1.46634969 |
| 118.07811__704.30 | Methyl 2-octynoate | 1.40E-07 | 24.698053 | 1.39266272 |
| 1109.78365__50.75 | Dilauryl 3,3'-thiodipropionate | 1.69E-10 | 15.863379 | 1.2003957 |
| 1109.78359__63.82 | Dilauryl 3,3'-thiodipropionate | 1.74E-09 | 78.860094 | 1.89685729 |
| 1109.78352__38.41 | Dilauryl 3,3'-thiodipropionate | 5.62E-10 | 44.028583 | 1.64373471 |
| 1064.45814__63.76 | Austalide D | 6.79E-10 | 27.237055 | 1.43516015 |
| 1064.45783__40.27 | Austalide D | 3.26E-09 | 28.96163 | 1.461823 |
| 1041.79498__58.30 | Methyl 3b-hydroxy-13(18)-oleanen-28-oate | 1.41E-09 | 45.663807 | 1.65957212 |
| 1007.80121__40.21 | TG(15:0/22:2(13Z,16Z)/22:6(4Z,7Z,10Z,13Z,16Z,19Z)) | 4.63E-09 | 81.575466 | 1.91155956 |
| 1007.80084__53.87 | TG(15:0/22:2(13Z,16Z)/22:6(4Z,7Z,10Z,13Z,16Z,19Z)) | 6.14E-09 | 20.38259 | 1.30925937 |
