# Supplementary material for: Circulating metabolites in patients with chronic heart failure are not related to gut leakage or gut dysbiosis
Source: PLoS One. 2025 Sep 8;20(9):e0331692. doi: 10.1371/journal.pone.0331692 (PMC12416712; doi:10.1371/journal.pone.0331692)
Supplement: S2 Table — (DOCX) [file pone.0331692.s003.docx]

**S2 Table.** Depleted metabolites annotation.

| Metabolites | Compound name | p-value | Odds ratio | Log(odds ratio) |
| --- | --- | --- | --- | --- |
| 110.08414__906.18 | D-Lysine | 9.30E-05 | 5.00E-06 | -5.30103 |
| 96.06878__1105.85 | Pyridine | 0.000191 | 1.30E-05 | -4.8860566 |
| 110.08416__956.25 | D-Lysine | 7.80E-05 | 2.90E-05 | -4.537602 |
| 82.05297__1079.17 | 2,4-Diaminobutyric acid | 0.000104 | 3.90E-05 | -4.4089354 |
| 127.11057__1156.36 | Piperazine | 5.00E-06 | 5.40E-05 | -4.2676062 |
| 127.11047__1049.01 | Piperazine | 9.41E-07 | 5.50E-05 | -4.2596373 |
| 111.08730__1285.98 | 1,1-Dimethylbiguanide | 1.67E-07 | 5.50E-05 | -4.2596373 |
| 127.11056__1225.78 | Piperazine | 5.00E-06 | 8.20E-05 | -4.0861861 |
| 127.11055__1241.21 | Piperazine | 1.00E-06 | 0.000138 | -3.8601209 |
| 82.05297__1123.85 | 2,4-Diaminobutyric acid | 7.00E-06 | 0.000169 | -3.7721133 |
| 96.06872__1153.11 | N-Methylene-ethenamine | 8.00E-06 | 0.00017 | -3.7695511 |
| 136.03759__534.94 | 1-Pentanesulfenothioic acid | 1.70E-05 | 0.000184 | -3.7351822 |
| 110.08415__772.90 | D-Lysine | 8.00E-06 | 0.0003 | -3.5228787 |
| 96.06869__1238.69 | Ornithine | 7.00E-06 | 0.000371 | -3.4306261 |
| 96.06867__1210.31 | Ornithine | 5.00E-06 | 0.000388 | -3.4111683 |
| 96.06867__1074.82 | Ornithine | 3.00E-06 | 0.000422 | -3.3746875 |
| 127.11045__1127.61 | Piperazine | 1.27E-07 | 0.000429 | -3.3675427 |
| 127.11054__1095.04 | Piperazine | 1.00E-06 | 0.000441 | -3.3555614 |
| 127.11029__1035.68 | Piperazine | 1.20E-05 | 0.000467 | -3.3306831 |
| 127.11041__1274.49 | Piperazine | 6.47E-07 | 0.000526 | -3.2790143 |
| 82.05293__1187.62 | 2,4-Diaminobutyric acid | 2.00E-06 | 0.000563 | -3.2494916 |
| 96.06873__1187.18 | 3-Amino-2-piperidone | 4.00E-06 | 0.000573 | -3.2418454 |
| 110.08428__815.37 | D-Lysine | 1.00E-06 | 0.000576 | -3.2395775 |
| 96.06870__1277.23 | Ornithine | 2.00E-06 | 0.000703 | -3.1530447 |
| 127.11041__944.84 | Piperazine | 6.00E-06 | 0.000781 | -3.107349 |
| 127.11053__1207.86 | Piperazine | 3.12E-07 | 0.000891 | -3.0501223 |
| 82.05301__1107.20 | 2,4-Diaminobutyric acid | 3.80E-07 | 0.000972 | -3.0123337 |
| 111.08728__1115.73 | 1,1-Dimethylbiguanide | 1.36E-06 | 0.00105 | -2.9788107 |
| 82.05295__1174.02 | 2,4-Diaminobutyric acid | 2.00E-06 | 0.00107 | -2.9706162 |
| 96.06872__1291.51 | N-Methylene-ethenamine | 2.00E-06 | 0.001178 | -2.9288547 |
| 110.08408__242.84 | D-Lysine | 3.10E-07 | 0.001205 | -2.919013 |
| 96.06864__1262.69 | Ornithine | 2.00E-06 | 0.001228 | -2.9108016 |
| 127.11050__1065.21 | Piperazine | 1.00E-06 | 0.001232 | -2.9093893 |
| 110.08417__89.00 | D-Lysine | 1.79E-07 | 0.001314 | -2.8814046 |
| 127.11048__1292.18 | Piperazine | 1.86E-07 | 0.001351 | -2.8693447 |
| 82.05297__1239.96 | 2,4-Diaminobutyric acid | 7.05E-07 | 0.001428 | -2.8452718 |
| 96.06877__1170.75 | 3-Amino-2-piperidone | 5.83E-07 | 0.001442 | -2.8410347 |
| 111.08727__1177.71 | 1,1-Dimethylbiguanide | 9.24E-08 | 0.001476 | -2.8309136 |
| 82.05297__1263.98 | 2,4-Diaminobutyric acid | 6.69E-07 | 0.00157 | -2.8041003 |
| 96.06874__1134.12 | 3-Amino-2-piperidone | 2.00E-06 | 0.001638 | -2.7856861 |
| 127.11057__906.84 | Piperazine | 2.00E-06 | 0.001998 | -2.6994045 |
| 82.05296__1280.27 | 2,4-Diaminobutyric acid | 1.53E-07 | 0.002113 | -2.6751005 |
| 127.11054__1187.21 | Piperazine | 1.81E-07 | 0.002143 | -2.6689778 |
| 110.08401__198.62 | D-Lysine | 3.35E-08 | 0.002319 | -2.6346993 |
| 96.06871__1122.10 | N-Methylene-ethenamine | 3.04E-07 | 0.002326 | -2.6333903 |
| 82.05301__1293.67 | 2,4-Diaminobutyric acid | 1.00E-07 | 0.00266 | -2.5751184 |
| 110.08424__794.40 | D-Lysine | 2.21E-07 | 0.002692 | -2.5699249 |
| 110.08427__324.52 | D-Lysine | 2.31E-08 | 0.002732 | -2.5635193 |
| 111.08735__1247.14 | 1,1-Dimethylbiguanide | 7.87E-08 | 0.003204 | -2.4943075 |
| 110.08447__106.71 | Aniline | 2.04E-07 | 0.003238 | -2.4897232 |
| 82.05296__1201.38 | 2,4-Diaminobutyric acid | 1.00E-06 | 0.003284 | -2.4835969 |
| 82.05297__1140.70 | 2,4-Diaminobutyric acid | 7.94E-08 | 0.003949 | -2.4035129 |
| 127.11052__1169.59 | Piperazine | 2.42E-07 | 0.004196 | -2.3771645 |
| 111.08746__1082.25 | 1,1-Dimethylbiguanide | 5.56E-07 | 0.004477 | -2.3490129 |
| 258.18291__511.59 | 5Z-Dodecenoic acid | 1.28E-07 | 0.004925 | -2.3075938 |
| 110.08423__215.11 | D-Lysine | 1.38E-08 | 0.005394 | -2.2680891 |
| 82.05298__1251.21 | 2,4-Diaminobutyric acid | 8.56E-08 | 0.00565 | -2.2479516 |
| 127.11052__1142.74 | Piperazine | 5.41E-08 | 0.005903 | -2.2289272 |
| 96.06859__579.61 | Ornithine | 9.21E-08 | 0.005996 | -2.2221384 |
| 82.05301__1092.90 | 2,4-Diaminobutyric acid | 7.42E-08 | 0.0061 | -2.2146702 |
| 82.05296__1156.63 | 2,4-Diaminobutyric acid | 1.48E-07 | 0.0061 | -2.2146702 |
| 127.11045__1110.36 | Piperazine | 6.30E-08 | 0.007914 | -2.101604 |
| 110.08420__1119.36 | D-Lysine | 2.26E-08 | 0.008546 | -2.0682371 |
| 127.11066__1007.68 | Piperazine | 7.91E-08 | 0.009729 | -2.0119318 |
| 110.08420__1079.23 | D-Lysine | 2.38E-08 | 0.009948 | -2.0022642 |
| 87.07956__1297.41 | Beta-Aminopropionitrile | 3.42E-08 | 0.011976 | -1.9216882 |
| 202.12030__511.59 | N-Lactoylisoleucine | 3.62E-08 | 0.012377 | -1.9073846 |
| 127.11040__1023.66 | Piperazine | 3.48E-07 | 0.012863 | -1.8906577 |
| 111.08718__1164.31 | 5,6,7,8-Tetrahydro-4-methylquinoline | 2.12E-07 | 0.013933 | -1.8559554 |
| 82.05295__1226.96 | 2,4-Diaminobutyric acid | 1.22E-07 | 0.014727 | -1.8318857 |
| 127.11054__979.36 | Piperazine | 1.84E-07 | 0.014986 | -1.8243143 |
| 111.08729__1151.12 | 1,1-Dimethylbiguanide | 1.27E-07 | 0.015058 | -1.8222327 |
| 127.11051__1080.36 | Piperazine | 3.33E-08 | 0.016349 | -1.7865088 |
| 110.08419__295.32 | D-Lysine | 7.25E-10 | 0.018947 | -1.7224595 |
| 110.08413__118.75 | D-Lysine | 1.19E-07 | 0.020849 | -1.6809148 |
| 110.08421__386.10 | D-Lysine | 1.50E-09 | 0.021966 | -1.658249 |
| 127.11057__1258.59 | Piperazine | 1.16E-08 | 0.02297 | -1.638839 |
| 127.11047__997.20 | Piperazine | 1.67E-07 | 0.024429 | -1.6120943 |
| 351.29825__531.85 | 20-Dihydrodydrogesterone | 2.04E-09 | 0.025294 | -1.5969825 |
| 110.08419__162.56 | D-Lysine | 1.09E-07 | 0.02538 | -1.5955084 |
| 110.08423__1004.56 | D-Lysine | 1.03E-09 | 0.025875 | -1.5871196 |
| 110.08420__871.74 | D-Lysine | 1.77E-09 | 0.027343 | -1.5631538 |
| 110.08413__74.47 | D-Lysine | 2.85E-09 | 0.029683 | -1.5274922 |
| 260.23491__560.81 | Tridecanol | 9.50E-08 | 0.030699 | -1.5128758 |
| 82.05298__1214.71 | 2,4-Diaminobutyric acid | 1.43E-09 | 0.034349 | -1.4640859 |
| 127.11040__880.08 | Piperazine | 1.23E-09 | 0.037021 | -1.4315519 |
| 110.08431__1048.43 | D-Lysine | 7.03E-10 | 0.037717 | -1.4234629 |
| 96.06861__341.25 | Ornithine | 1.00E-06 | 0.040255 | -1.3951802 |
| 172.14698__86.32 | Capric acid | 5.31E-10 | 0.050701 | -1.2949835 |
| 111.08744__1098.41 | 1,1-Dimethylbiguanide | 1.98E-07 | 0.056236 | -1.2499856 |
| 127.11055__1198.74 | Piperazine | 1.07E-09 | 0.059268 | -1.2271797 |
| 127.11036__932.19 | Piperazine | 3.87E-10 | 0.060903 | -1.2153613 |
| 96.06791__374.36 | Ornithine | 3.18E-07 | 0.063186 | -1.1993791 |
| 103.09953__628.69 | Iso-Valeraldehyde | 3.27E-10 | 0.066808 | -1.1751715 |
| 164.08343__386.51 | Amyl 2-furoate | 9.72E-09 | 0.067277 | -1.1721334 |
| 110.08422__1176.62 | D-Lysine | 8.47E-10 | 0.067605 | -1.1700212 |
| 116.08403__141.18 | Caproic acid | 5.28E-10 | 0.073758 | -1.1321909 |
| 111.06845__1187.96 | N-Acetyl-2,3-dihydro-1H-pyrrole | 2.00E-06 | 0.074929 | -1.1253501 |
| 134.09407__198.13 | 1-Butanol | 2.96E-09 | 0.082917 | -1.0813564 |
| 82.05298__1167.32 | 2,4-Diaminobutyric acid | 1.99E-09 | 0.08371 | -1.0772227 |
| 401.32959__663.40 | Vitispirane | 1.11E-10 | 0.086914 | -1.0609103 |
| 118.07817__386.52 | Methyl 2-octynoate | 9.76E-10 | 0.088118 | -1.0549354 |
| 102.06863__166.58 | Isovaleric acid | 4.02E-10 | 0.090236 | -1.0446202 |
| 110.08418__1296.45 | D-Lysine | 3.55E-10 | 0.09091 | -1.0413883 |
| 84.06849__1165.32 | 2-(Dimethylamino)acetonitrile | 3.00E-06 | 0.092336 | -1.0346289 |
| 186.06529__386.51 | 4-Isopropylbenzoic acid | 4.92E-10 | 0.093867 | -1.0274871 |
| 218.03791__433.45 | Hypotaurine | 3.51E-09 | 0.094738 | -1.0234758 |
| 156.07713__215.14 | Polypropylene glycol (m w 1,200-3,000) | 2.60E-09 | 0.095517 | -1.0199193 |
| 144.11549__140.45 | Caprylic acid | 1.74E-11 | 0.095632 | -1.0193968 |
| 110.08427__1311.30 | D-Lysine | 2.36E-09 | 0.099182 | -1.0035671 |
| 294.25559__681.86 | Heptadecanoic acid | 1.25E-09 | 0.099774 | -1.0009826 |
