# Supplementary material for: Circulating metabolites in patients with chronic heart failure are not related to gut leakage or gut dysbiosis
Source: PLoS One. 2025 Sep 8;20(9):e0331692. doi: 10.1371/journal.pone.0331692 (PMC12416712; doi:10.1371/journal.pone.0331692)
Supplement: S3 Table — (DOCX) [file pone.0331692.s004.docx]

**S3 Table.** Enriched lipids annotation.

| Lipids | Compound name | p-value | Odds ratio | Log(odds ratio) |
| --- | --- | --- | --- | --- |
| 637.56417__490.28 | DG(14:0/20:0/0:0) | 2.25E-07 | 209.124105 | 2.3204041 |
| 301.29789__257.75 | Sphinganine | 6.00E-06 | 50.574318 | 1.70393003 |
| 357.36042__312.43 | Behenic acid | 4.00E-06 | 43.163321 | 1.63511485 |
| 557.46376__392.39 | TG(8:0/8:0/a-13:0)[rac] | 7.03E-09 | 40.600281 | 1.60852904 |
| 329.32913__287.40 | Octadecylamine | 5.00E-06 | 36.575495 | 1.56319021 |
| 273.26656__211.29 | Palmitic acid | 2.00E-06 | 35.931074 | 1.5554702 |
| 903.82398__619.16 | TG(14:0/24:0/14:0) | 5.15E-09 | 33.962876 | 1.53100446 |
| 429.90640__60.21 | - | 1.94E-09 | 31.520462 | 1.49859257 |
| 901.80830__606.78 | Glycerol 1-(9Z-octadecenoate) 2-hexadecanoate 3-octadecanoate | 1.53E-09 | 27.454858 | 1.4386192 |
| 565.88121__60.21 | Perfluorooctanesulfonic acid | 1.98E-09 | 24.438585 | 1.38807606 |
| 931.85423__608.05 | TG(18:0/18:0/18:0) | 6.59E-10 | 18.364723 | 1.26398438 |
| 515.41833__392.42 | Arachidyl carnitine | 1.84E-10 | 18.238686 | 1.26099355 |
| 929.83885__596.42 | Glycerol 1,3-dioctadecanoate 2-(9Z-octadecenoate) | 5.92E-10 | 14.984722 | 1.17564869 |
| 917.83094__608.06 | TG(14:0/15:0/24:0) | 8.56E-10 | 14.311913 | 1.15569769 |
| 767.54643__452.76 | PS(15:0/22:1(13Z)) | 1.22E-09 | 14.134967 | 1.1502948 |
| 623.63024__480.86 | Ficin | 5.19E-09 | 14.118421 | 1.14978613 |
| 637.64791__481.05 | - | 7.47E-09 | 13.853308 | 1.14155349 |
| 875.79285__608.11 | TG(18:0/14:0/18:0) | 9.65E-10 | 13.574519 | 1.13272445 |
| 899.79206__595.28 | TG(18:1(11Z)/16:0/18:1(11Z)) | 8.23E-08 | 13.116899 | 1.11783117 |
| 330.27663__323.76 | Heptadecanoic acid | 3.00E-06 | 12.355089 | 1.09184588 |
| 358.30808__344.00 | Nonadecanoic acid | 4.00E-06 | 11.854066 | 1.07386734 |
| 635.54854__469.45 | DG(14:0/20:1(11Z)/0:0) | 4.04E-05 | 11.279179 | 1.05227749 |
| 414.20412__213.14 | Melleolide B | 1.90E-05 | 11.052682 | 1.04346767 |
