# Supplementary material for: Circulating metabolites in patients with chronic heart failure are not related to gut leakage or gut dysbiosis
Source: PLoS One. 2025 Sep 8;20(9):e0331692. doi: 10.1371/journal.pone.0331692 (PMC12416712; doi:10.1371/journal.pone.0331692)
Supplement: S4 Table — (DOCX) [file pone.0331692.s005.docx]

**S4 Table.** Depleted lipids annotation.

| Lipids | Compound name | p-value | Odds ratio | Log(odds ratio) |
| --- | --- | --- | --- | --- |
| 334.27177__256.60 | 20-Dihydrodydrogesterone | 4.00E-06 | 0.004851 | -2.3141687 |
| 290.24552__256.52 | Dodecanal dimethyl acetal | 1.00E-06 | 0.005985 | -2.2229358 |
| 246.21936__255.80 | Dodecanol | 5.75E-07 | 0.006348 | -2.1973631 |
| 457.17358__218.14 | 3,4,5-trihydroxy-6-[3-methoxy-4-(3-phenylprop-2-en-1-yl)phenoxy]oxane-2-carboxylic acid | 4.33E-07 | 0.006527 | -2.1852864 |
| 395.32463__256.43 | 5alpha-Tomatidan-3-one | 3.19E-07 | 0.007445 | -2.1281353 |
| 411.37100__380.86 | Tricosanoylglycine | 1.98E-07 | 0.008939 | -2.0487111 |
